# Ocean temperatures through the Phanerozoic reassessed

**DOI:** 10.1038/s41598-022-11493-1

**Published:** 2022-05-27

**Authors:** Ethan L. Grossman, Michael M. Joachimski

**Affiliations:** 1 Department of Geology and Geophysics, Texas A&M University, College Station, TX 77843 USA; 2 GeoZentrum Nordbayern, Friedrich-Alexander Universität of Erlangen-Nürnberg (FAU), Schlossgarten 5, 91054 Erlangen, Germany

**Keywords:** Palaeoceanography, Palaeoclimate

## Abstract

The oxygen isotope compositions of carbonate and phosphatic fossils hold the key to understanding Earth-system evolution during the last 500 million years. Unfortunately, the validity and interpretation of this record remain unsettled. Our comprehensive compilation of Phanerozoic δ^18^O data for carbonate and phosphate fossils and microfossils (totaling 22,332 and 4615 analyses, respectively) shows rapid shifts best explained by temperature change. In calculating paleotemperatures, we apply a constant hydrosphere δ^18^O, correct seawater δ^18^O for ice volume and paleolatitude, and correct belemnite δ^18^O values for ^18^O enrichment. Similar paleotemperature trends for carbonates and phosphates confirm retention of original isotopic signatures. Average low-latitude (30° S–30° N) paleotemperatures for shallow environments decline from 42.0 ± 3.1 °C in the Early-to-Middle Ordovician to 35.6 ± 2.4 °C for the Late Ordovician through the Devonian, then fluctuate around 25.1 ± 3.5 °C from the Mississippian to today. The Early Triassic and Middle Cretaceous stand out as hothouse intervals. Correlations between atmospheric CO_2_ forcing and paleotemperature support CO_2_’s role as a climate driver in the Paleozoic.

## Introduction

The ^18^O/^16^O ratios of biogenic carbonate and apatite are an essential proxy for paleotemperature and seawater δ^18^O. For decades, however, Earth scientists have debated the climate implications of the Phanerozoic ^18^O/^16^O record (reported as δ^18^O), which shows increasing values with decreasing age (e.g. Refs.^1–4^). The increase in biomineral δ^18^O toward the present has been interpreted three ways: (1) cooling of the Earth’s oceans through time (e.g. Ref.^5^), (2) evolving crustal cycling manifested in an increase in seawater δ^18^O (δ^18^O_sw_; e.g. Refs.^2,3^), and (3) progressive sample diagenesis with age (e.g. Ref.^6^). Correct interpretation of this record is fundamental to understanding climate drivers, the limits of Earth’s climate, and importantly, the evolution and thermal limits of metazoan life on geologic timescales.

To characterize and evaluate the Phanerozoic trend in biomineral δ^18^O and sea surface temperature (SST), we present a compilation of low-latitude (30° S–30° N) δ^18^O paleotemperatures for Phanerozoic carbonate and phosphate fossils and microfossils that, in contrast to earlier published records, corrects for the influence of ice volume and salinity (via paleolatitude relationships) on the δ^18^O_sw_ of surface waters. Furthermore, for the first time we consider the impact of fractionation differences in belemnites^7,8^ to develop a 500-million-year paleotemperature curve that resolves the problem of anomalously cold paleotemperatures previously obtained for intervals in the Jurassic and Cretaceous. The compiled data reveal similar trends for carbonate and phosphate δ^18^O, providing evidence for signal preservation, and argue for extreme warmth in the early Paleozoic (30 to > 40 °C), comparable to that experienced at the end-Permian and earliest Triassic^9,10^.

## Results

### Phanerozoic oxygen isotope records

**Figure 1:**
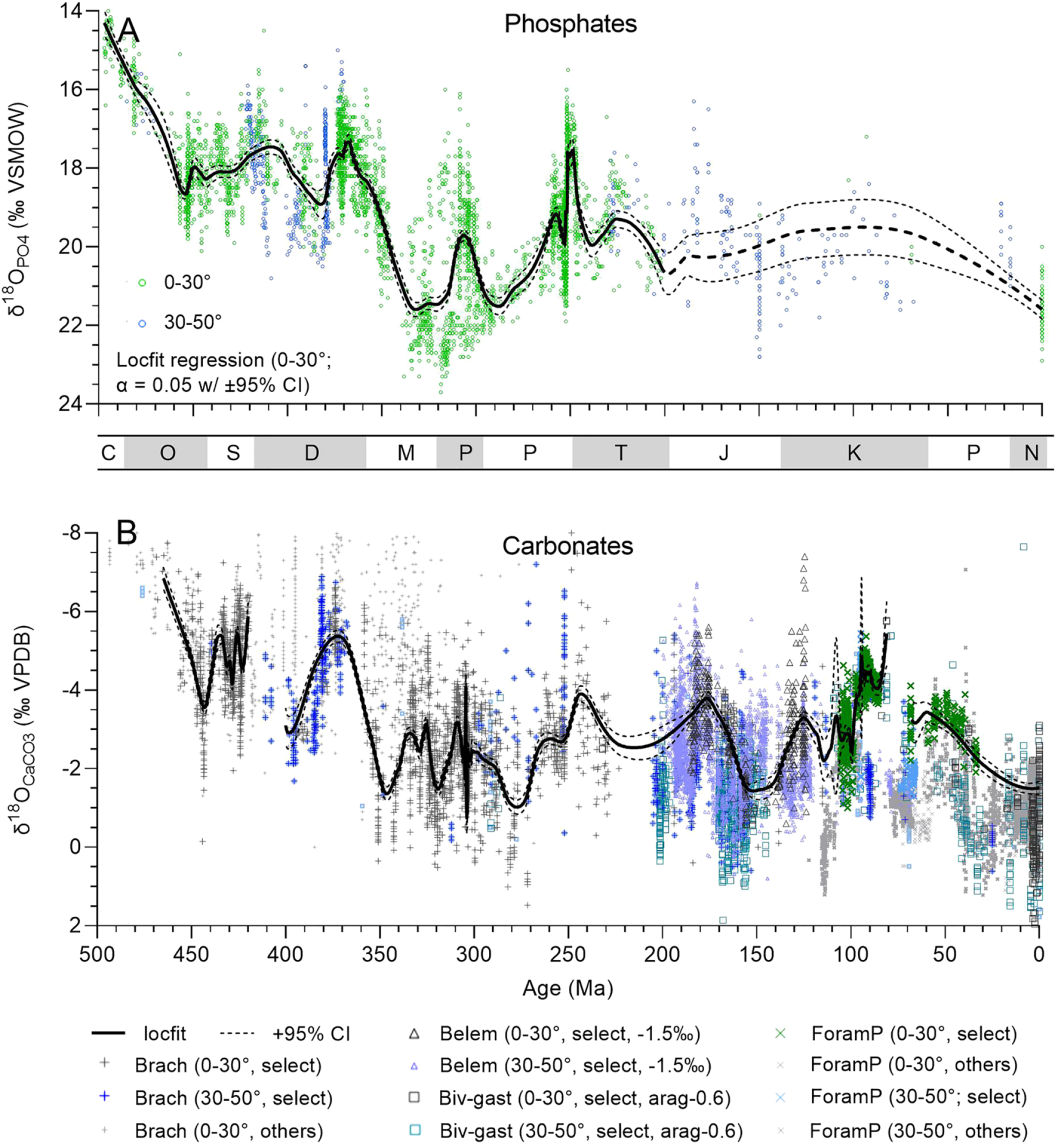
Oxygen isotope compositions of phosphate (**A**) and carbonate fossils and microfossils (**B**) from tropical-subtropical (0–30°) and temperate (30–50°) latitudes. Carbonate δ^18^O values less than −8‰ (N = 139) are not shown. These are almost exclusively Paleozoic brachiopod shells that fail quality control (QC) standards (i.e., not “select”). Locfit regression lines (α = 0.05) for tropical and subtropical samples are shown with ± 95% confidence limits (CL). ForamP = planktonic foraminifera, Biv-gast = bivalves and gastropods, Belem = belemnites, Brach = brachiopods, and select = data that have passed QC standards. Belemnite values were adjusted by −1.5‰ to correct for ^18^O enrichment. Letters in the bar between figures refers to geologic periods Cambrian, Ordovician, Silurian, Devonian, Mississippian, Pennsylvanian, Permian, Triassic, Jurassic, Cretaceous, Paleogene, and Neogene. Some symbols in key are enlarged for clarity.

The carbonate δ^18^O record combines data for well-preserved planktonic foraminifera, mollusks, and brachiopods (Fig. 1B). The data for well-preserved carbonates (N = 11,893 of 22,332 total) and phosphates (N = 4,427 of 4615 total) are available in Appendices 1 and 2 and the StabisoDB database (http://stabisodb.org). Counts of δ^18^O analyses by stage, fossil group, and climate zone are in Appendix 3. The Locfit regressions (Locfit package in R version 3.6.2; smoothing factor α = 0.05^11^) combine data for the tropical (10° S–10° N) and tropical-subtropical (10–30° N and S latitude) climate zones. Distinctive features of the Phanerozoic record for carbonates include extremely low brachiopod δ^18^O values (~ −7.5‰ VPDB) for the Middle Ordovician, a latest Ordovician (Hirnantian) maximum (−3.6‰), an Early Devonian minimum (−5.9‰), a Middle Devonian maximum (−2.9‰), and a Late Devonian (Frasnian–Famennian) minimum (−5.2‰). Average brachiopod values increase to a Mississippian maximum of −1.1‰ and fluctuate between −3.3 and −1.2‰ in the Pennsylvanian and Early Permian before decreasing into the Triassic (< −3‰), a period for which carbonate δ18O data are scarce. For the Jurassic and Cretaceous, periods for which belemnite data dominate, the δ18O record features high values in the Late Jurassic (−1.5‰, Oxfordian-Kimmeridgian) and Early Cretaceous (−2.2‰, Aptian), when the foraminiferal record begins. Values then decrease to an early Late Cretaceous minimum (−5.6‰, Turonian), followed by an irregular increase in the Neogene to modern values (−1.9 ± 0.3‰).

**Figure 2:**
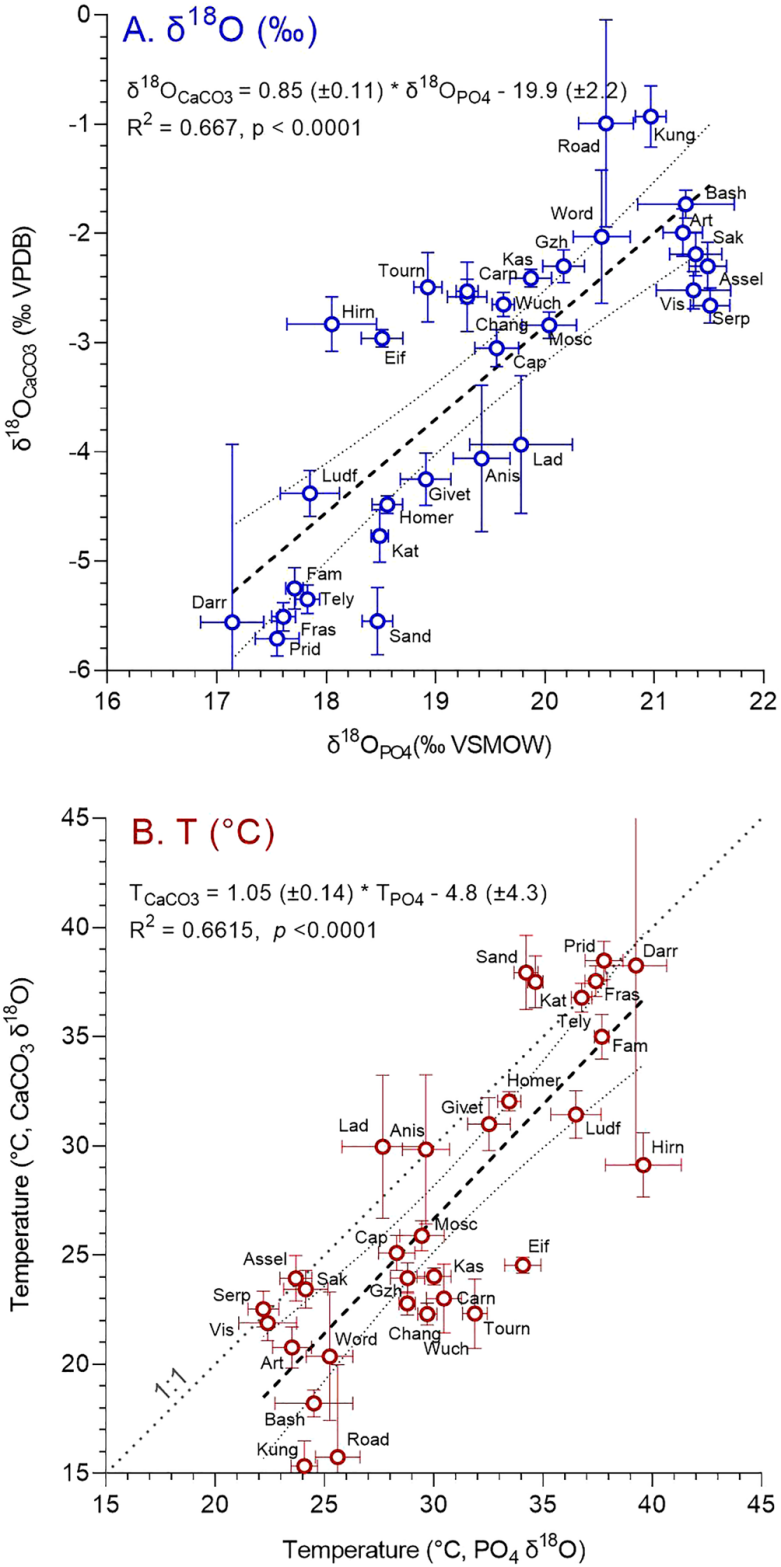
Comparison of low-latitude (30° S to 30° N) δ^18^O values (**A**) and δ^18^O paleotemperatures (**B**) of phosphate and carbonate fossils and microfossils of Paleozoic and Triassic ages averaged by stage. The regression line was generated using a simple linear regression model because of the lower uncertainty in phosphate versus carbonate values (see Supplementary Information). Note that the slope for δ^18^O_PO4_ versus δ^18^O_CaCO3_ (0.85) is nearly identical to the slope of the phosphate-carbonate paleotemperature relations (0.82), and the slope for T_PO4_ versus T_CaCO3_ is 1.05. Both correlations are significant (*p* < 0.0001).

Key features in the conodont δ^18^O record (Fig. 1A) mimic those of the carbonate record. Conodont δ^18^O values increase from very low values in the Early Ordovician (< 17‰ VSMOW) to higher values (19.0‰) in the Late Ordovician (Sandbian), followed by a minimum (17.5‰) in the earliest Silurian (Llandovery), a Wenlock maximum (18.5‰), and an Early Devonian minimum (17.1‰; Lochkovian). Values increase during the Early Devonian to a Middle Devonian maximum (~ 19.3‰) followed by a minimum (17.2‰) at the Frasnian-Famennian transition. After a Late Devonian high (18.3‰), values increase substantially during the Mississippian. Oxygen isotope compositions of Pennsylvanian conodonts from epicontinental (e.g., North America) and slope settings (South China) show considerable offset (~ 19.5‰ versus ~ 23‰ respectively) potentially due to lower salinities in shallow-water North American settings or deeper habitat depths of South China conodonts. The δ^18^O values decrease sharply from ~ 20.4 to ~ 17.7‰ across the Permian–Triassic boundary with low δ^18^O values persisting in the Early Triassic. In contrast to the Cambrian to Triassic conodont δ^18^O record, the Cenozoic fish δ^18^O record is of too low resolution to allow detailed interpretation. Direct comparison of stage averages (Fig. 2A) shows a strong correlation between carbonate and phosphate δ^18^O values (R^2^ = 0.667, *p* < 0.0001). The slope of 0.85 is nearly identical to the slope (0.82) of “equilibrium” carbonate δ^18^O (‰ VPDB) versus phosphate δ^18^O (‰ VSMOW) over the temperature range 10 to 45 °C for the paleotemperature equations used^12,13^, supporting the integrity of these records.

### Phanerozoic low-latitude temperatures

**Figure 3:**
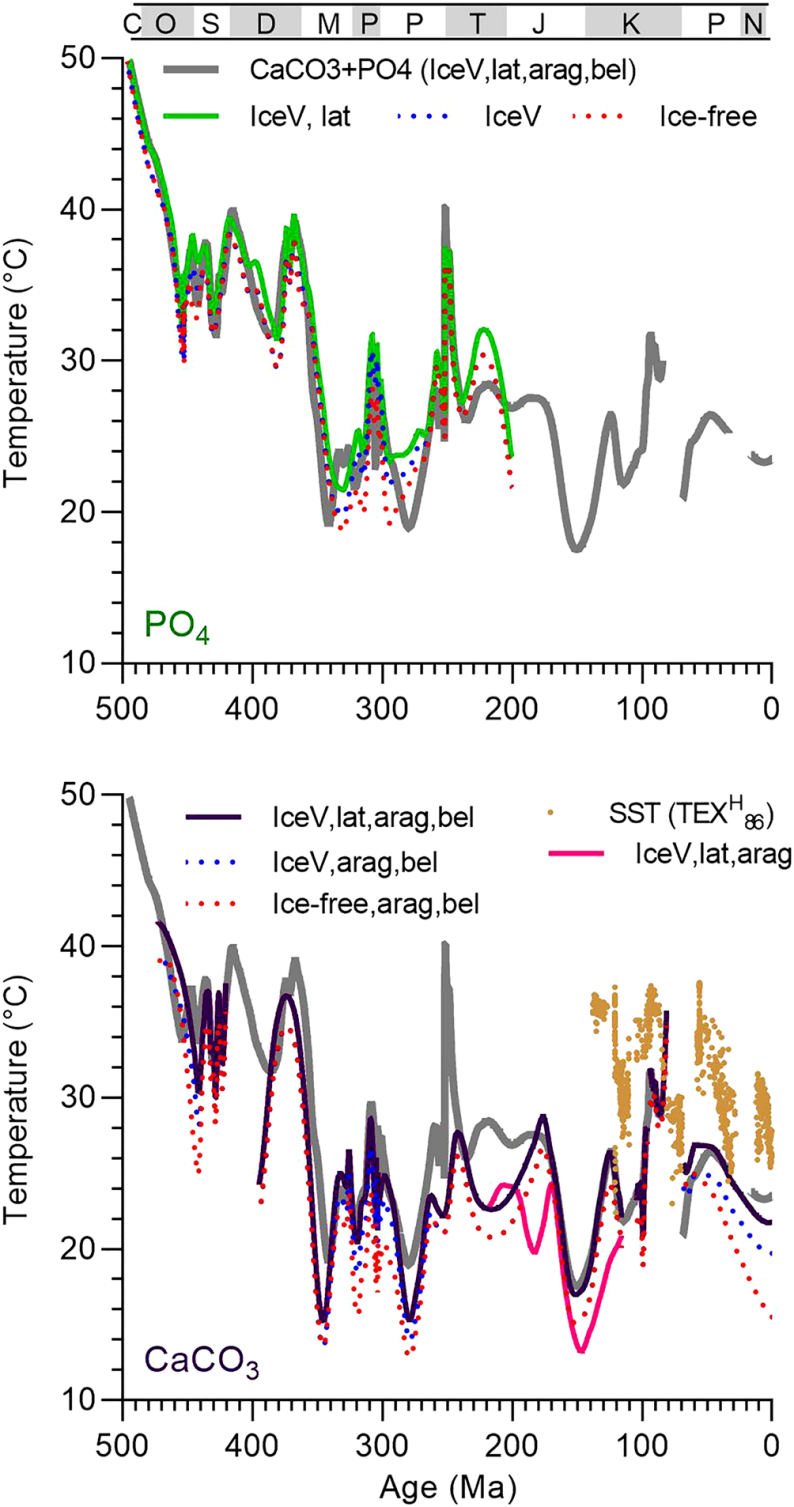
δ^18^O paleotemperatures for phosphate and carbonate fossils and microfossils from low paleolatitudes (30° S to 30° N). Comparison of δ^18^O paleotemperature trends (Locfit regressions) for phosphate and carbonate fossils calculated using different corrections for seawater δ^18^O: (1) constant (ice-free, δ^18^O_sw_ = −1.08‰ VSMOW), (2) ice volume corrected (IceV), and (3) ice volume and latitude corrected (IceV, lat). For greenhouse climates, the ice-free data (red dotted line) plot atop the ice volume corrected data (blue dotted lines). Aragonite fossils are corrected for aragonite-calcite fractionation (arag; −0.6‰) and belemnite data are corrected for ^18^O enrichment in belemnites (−1.5% where noted (bel). Locfit regression for data uncorrected for ^18^O enrichment in belemnites shown as pink line. Gaps in curves are intervals with little or no data. TEX^H^_86_ temperatures shown for comparison^16,17^. See Fig. 1 for key to period/subperiod bar at top.

**Figure 4:**
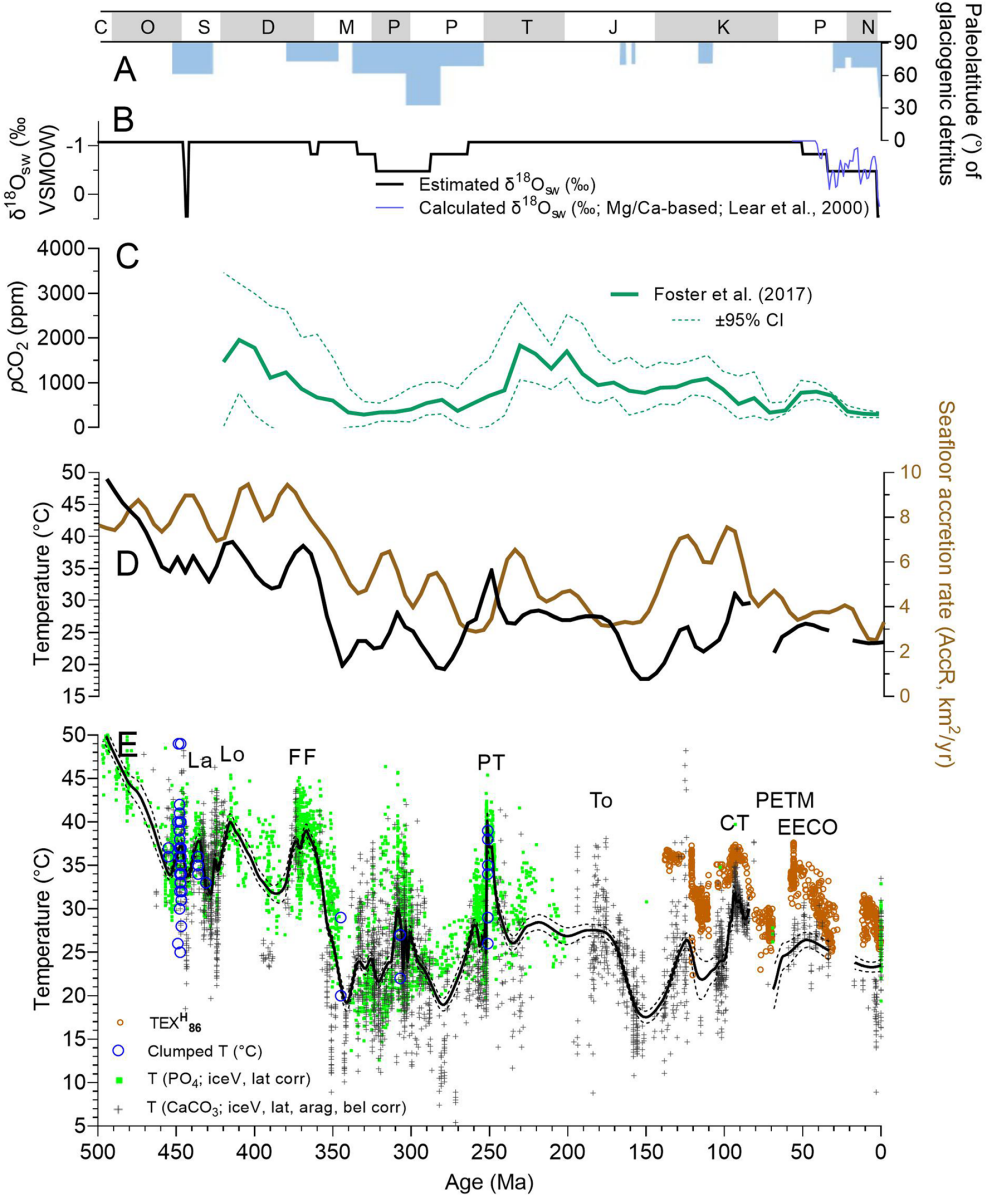
Comparison of oxygen isotope temperatures (E) with climate and tectonic proxy data. (**A**) Occurrence and latitudinal extent of glaciogenic sediments^20^; (**B**) estimated δ^18^O_sw_ (this study); (**C**) atmospheric *p*CO_2_^21^; (**D**) crustal accretion rate^22^ compared with combined phosphate and carbonate δ^18^O paleotemperature (low-latitude, Locfit regression and 5-myr averages); (**E**) Low-latitude phosphate and carbonate δ^18^O paleotemperatures calculated by correcting seawater δ^18^O for ice volume (iceV) and latitude (lat). Locfit regression (α = 0.05) with 95% Cl calculated for combined carbonate and phosphate δ^18^O paleotemperatures. Paleozoic clumped isotope paleotemperatures (clumped T) from Henkes et al.^23^ and Barney and Grossman^24^. Also shown are TEX_86_ paleotemperatures using the TEX_86_^H^-SST calibration^16,17^. Geologic time scale abbreviations same as in Fig. 1. Warm events: *La* Landovery, *Lo* Lochkovian, *FF* Frasnian–Famennian, *PT* end-Permian and early Triassic, *To* Toarcian, *CT* Cenomanian–Turonian, *PETM* Paleocene-Eocene Thermal Maximum, *EECO* Early Eocene Climatic Optimum.

Paleozoic paleotemperatures based on carbonates and phosphates (Figs. 3, 4) show similar trends such as very high late Cambrian and Early to Middle Ordovician temperatures (> 40 °C), high early Late Ordovician through Devonian temperatures (32–40 °C), a dramatic decline of as much as 15 °C at the Devonian to Mississippian transition, and cooler temperatures in the Carboniferous and Permian (19–35 °C; Fig. 4). The records show disagreement at intervals in which brachiopods were derived from paleo-arid regions, where high δ^18^O_sw_ values, underestimated by our model, result in underestimated isotopic temperatures. Examples include Tournaisian-Viséan data from Indiana (USA) and Kungurian-Roadian data from the Ural Mountains (Russia)^14,15^. Paleotemperature differences also occur because of differences in the distribution of sample ages. Whereas brachiopods provide a robust record of the Hirnantian Cool Event, conodont data are essentially absent. In contrast, the hothouse at the end-Permian and Early Triassic is well represented in conodont data but not in brachiopod data^9,10^.

Simple linear regression of carbonate and phosphate δ^18^O paleotemperatures averaged for Paleozoic and Triassic stages (Fig. 2B, Supplementary Tables 1 and S1) yields an equation:

1 $${\text{T}}_{{{\text{PO4}}}} = { 1}.0{5 }\left( { \pm 0.{14}} \right){\text{ T}}_{{{\text{CaCO3}}}} {-}{ 4}.{8 }\left( { \pm {4}.{3};{\text{ R}}^{{2}} = \, 0.{6615},p < 0.000{1}} \right).$$

The strong correlation (R^2^ = 0.6615) and slope near 1 are evidence that these materials retained their original isotopic signature. The ~ 4 °C average offset from the 1:1 line suggests higher paleotemperatures for the phosphate samples. This difference could reflect differences in aridity and the spatial and temporal distributions of specimens as mentioned above (e.g. Ref.^18^). Another contributing factor may be depth habitat. At some locations, nektonic conodonts may have lived shallower in the water column than benthic brachiopods. Lastly, differences in paleotemperature may indicate uncertainties in the paleotemperature equations. Applying the equation of Friedman and O’Neil^19^ for calcite decreases the temperature difference between Paleozoic stages from 3.6 to 2.0 °C. However, we use the Kim and O’Neil^12^ equation because it is more widely applied by the paleoclimate community. Combining the results for conodonts and brachiopods provides a continuous and comprehensive paleotemperature record for low latitudes (30° S–30° N; Fig. 4). Late Cambrian (Furongian) and Early to Middle Ordovician low-latitudes experienced the highest SSTs, 47 °C and between 35 and 47 °C, respectively. Late Ordovician to Devonian low-latitude SSTs were considerably cooler (32–40 °C). Cooler still were Carboniferous to modern low-latitude SSTs, which varied between 19 and 35 °C with relatively cool “icehouse” temperatures observed in the Carboniferous to Middle Permian (19–24 °C), Middle Jurassic to Early Cretaceous (18–27 °C), and late Eocene to modern times (23–25 °C). The late Permian to Middle Jurassic and Late Cretaceous to Eocene are recognized as warm climatic intervals (25–30 °C) with the Permian–Triassic transition, Early Triassic, and Cenomanian–Turonian standing out as hothouse intervals. The Paleocene-Eocene Thermal Maximum (PETM), another very warm period, is not well represented in our oxygen isotope dataset due to insufficient data for shallow-dwelling organisms.

## Discussion

The decrease in marine fossil δ^18^O with age, the trend expected with oxygen isotope exchange with meteoric water, has led some to question the preservation and efficacy of the original marine δ^18^O signal, especially with regard to the Paleozoic record. We address these concerns by restricting Paleozoic samples to the best-preserved material, brachiopod shell calcite and conodont apatite. Phosphate oxygen is more tightly bound in the crystal lattice than is calcite oxygen; in fact, apatite phosphate survives dissolution and reprecipitation as trisilverphosphate during sample preparation without altering its oxygen isotope composition. That both minerals yield similar δ^18^O trends through time (Figs. 2, 3) is evidence for preservation of primary signals. Additional evidence is comparable δ^18^O values for Pennsylvanian calcite versus aragonite^25^ and for Devonian conodont apatite experiencing minimal versus extensive heating^26^.

**Figure 5:**
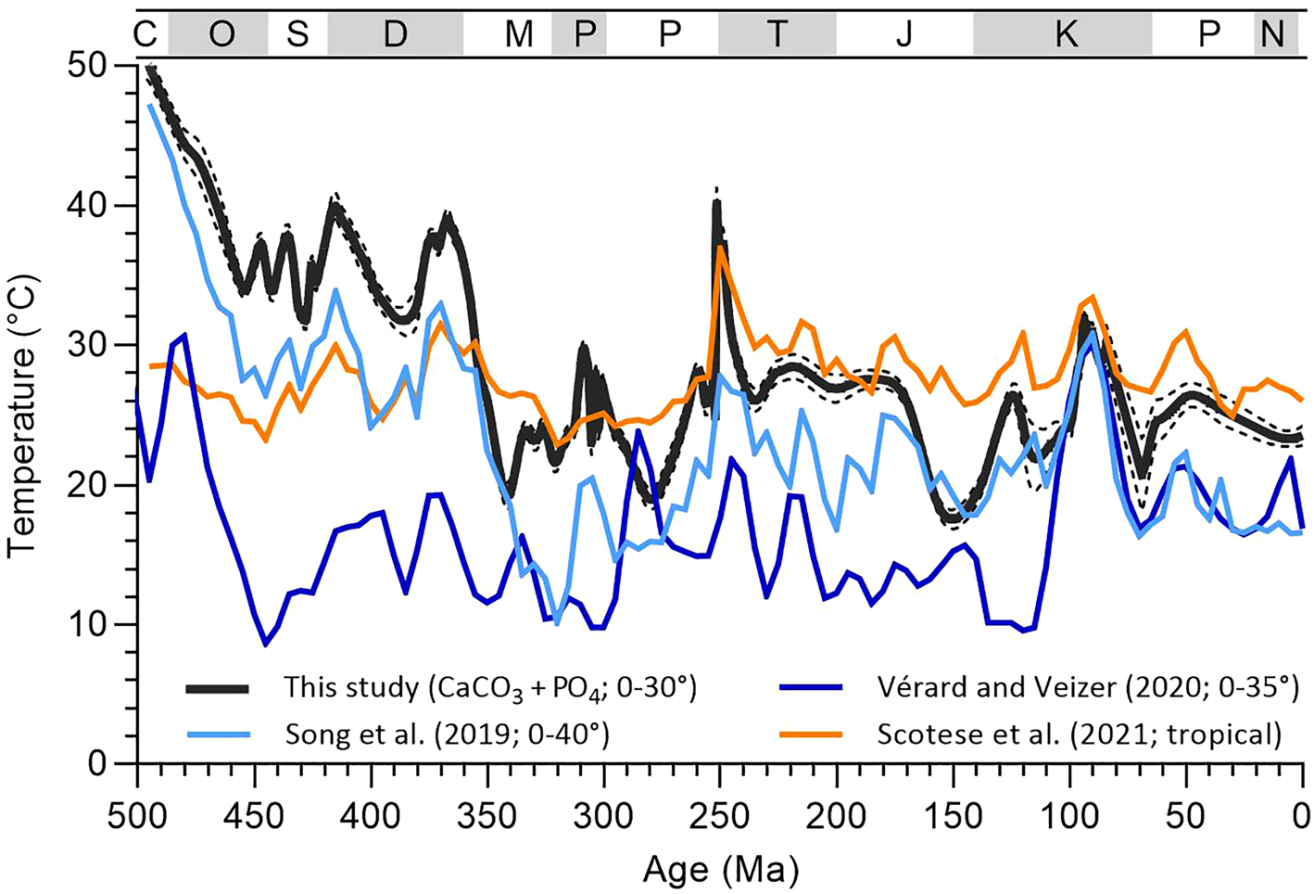
Comparison of low-latitude Phanerozoic temperature curves from this study (30° S to 30° N), Song et al. (2019; 40° S to 40° N), Vérard and Veizer (2020; 35° S to 35° N), and Scotese et al.^30^ (“tropical”). Differences in curves reflect in part acceptance (this study; Song et al.^30^) or rejection^22,30^ of the buffered hydrosphere δ^18^O model^31,32^ which constrains global seawater δ^18^O between roughly −1 and 1‰ VSMOW. Geologic time scale abbreviations same as in Fig. 1. Dashed lines represent ± 95% confidence interval for this study’s results.

As with previous studies, our δ^18^O results show an increase from very low late Cambrian through Middle Ordovician values toward higher δ^18^O values in the modern (Fig. 5). Not surprisingly, our paleotemperatures show many of the same relative changes as in previous studies, an expectation considering the overlap in data sources; however, substantial differences occur in absolute temperature with higher Early Paleozoic temperatures in our study and much lower Phanerozoic temperatures in other studies. These offsets reflect differences in (1) sample material (carbonates versus combined carbonates and phosphates), (2) screening approaches, (3) methods of estimating seawater δ^18^O, and (4) paleotemperature equations^1,4,27–30^.

We start the discussion with Veizer and Prokoph’s^4^ seminal work because several studies adopt their data and interpretation (e.g., Refs.^22,33^). With regard to sample material, the Veizer and Prokoph^4^ δ^18^O curve is based on data only from brachiopods and planktonic foraminifera and excludes data from phosphates (e.g., conodonts), belemnites, bivalves, and gastropods. Importantly, planktonic foraminifera can be readily recrystallized on the cold sea floor; specimens not characterized as glassy or excellently preserved can yield δ^18^O values 1‰ higher than glassy foraminifera^34^. This in part accounts for very low paleotemperatures for the Cenozoic and Cretaceous. For example, Vérard and Veizer obtained average low-latitude paleo-SSTs of 10 °C for the Early Cretaceous (115–135 Ma), a time of greenhouse climate.

Another reason for unusually low paleotemperatures in Veizer and Prokoph^4^ and Vérard and Veizer^22^ is the assumption regarding seawater δ^18^O. Interpreting the δ^18^O trend as reflecting changes in seawater δ^18^O, they (1) fit a 2^nd^ order regression to the trend, (2) adjust the equation to intersect δ^18^O = 0‰ VPDB at 0 Ma (i.e., set Y-intercept at 0‰), and (3) use the regression to “correct” carbonate δ^18^O values for changes in δ^18^O_sw_. The effect of this treatment is to set modern low-latitude SST for the dataset at ~ 18.0 °C, more than 7.4 °C lower that the modern average (25.4 °C; https://psl.noaa.gov/data/gridded/data.cobe.html) for that study’s paleolatitude window (35° S to 35° N) and 6.5 °C lower than average tropical proxy temperatures for the Late Pleistocene (~ 24.5 °C^35^). By comparison, for our study interval (30° S to 30° N) the temperature at 0 Ma is 23.5 °C, much closer to the modern and Late Pleistocene values. Veizer and Prokoph’s^4^ age correction of δ^18^O_sw_ compensates for the low mineral δ^18^O values for the Ordovician through Devonian, shifting biomineral δ^18^O values + 5 to + 2‰ respectively, equivalent to 22 to 9 °C. A third consideration is the paleotemperature equation. Veizer and Prokoph^4^ and Mills et al.^33^ use a linear equation with a δ^18^O-temperature dependence of −4 °C per ‰, significantly lower than that of other studies (−4.3 to −4.8 °C per ‰; Grossman^28^), resulting in paleotemperature underestimation at temperatures above ~ 25 °C. These factors contribute to these studies’ findings of equable low-latitude paleotemperatures for Early Paleozoic oceans, and to the excessively cold temperatures of 10–12 °C for Pennsylvanian and early Permian oceans (325–295 Ma).

Another important Phanerozoic temperature curve is that of Song et al.^29^. This curve yields lower temperatures in the Paleozoic, early Mesozoic, and Cenozoic compared with ours (Fig. 5). These authors make use of phosphate δ^18^O data for the Paleozoic and carbonate δ^18^O data for the Mesozoic and Cenozoic, noting the better δ^18^O preservation of apatite compared with the calcite in Paleozoic fossils. These authors reject the hypothesis of increasing seawater δ^18^O in the Phanerozoic^4^ and instead assume a constant seawater δ^18^O of −1‰ VSMOW, representing ice-free conditions. The lower temperatures in Song et al.^29^ reflect (1) use of an ice-free seawater δ^18^O value during glacial conditions (an up to −6 °C effect; e.g., 10 °C temperatures for mid-Carboniferous), (2) lack of paleolatitude corrections for seawater δ^18^O (an effect of up to −5 °C for the subtropics), and (3) use of the phosphate-water paleothermometers of Lécuyer et al. (2013; an effect of up to −3.5 °C). We use the Pucéat et al.^13^ equation, which was produced in the same lab where the majority of Paleozoic phosphate samples were analyzed. Song et al.^29^ also includes samples for higher latitudes (up to ± 40°), which could lower paleotemperatures. However, in StabisoDB, phosphate samples from 30 to 40° paleolatitude only represent 7.5% of the samples between 0 and 40° paleolatitude, so this effect should be minor. Scotese et al.’s^30^ Phanerozoic temperature curve shows less extreme temperatures in the Early Paleozoic and late Cenozoic (Fig. 5). The curve uses a combination of isotopic data^29^ and paleotemperatures based on paleo-Köppen climate belts calibrated with modern Köppen belt temperature relations. While novel, this hybrid paleotemperature curve implicitly assumes modern temperature relations and thus may inject a uniformitarian bias into quantification of Earth’s temperature history. As discussed earlier, our Phanerozoic curve is the first to correct δ^18^O_sw_ for paleolatitude and for ^18^O-enrichment in belemnites, which accounts for warmer proxy temperatures for the Jurassic compared with other isotopic studies.

Accepting that the Phanerozoic δ^18^O trend is not an artifact of diagenesis, the debate as to whether the trend reflects δ^18^O_sw_ or temperature change distills to two endmember assumptions: (1) no long-term trend in seawater δ^18^O (e.g. Ref.^36^) and (2) no long-term trend in low-latitude SST^3,4,27^. In theory, the oxygen isotope history of seawater can be calculated from the rates and temperatures of oxygen exchange between the mantle, crustal reservoirs, and the ocean. Mass balance models have been used to argue for near-constant (e.g. Refs.^36–38^) or increasing (e.g. Refs.^39,40^) δ^18^O_sw_, depending on the proportion of crustal oxygen exchange at high-temperature (which increases δ^18^O_sw_) versus low-temperature (which decreases δ^18^O_sw_). Models that predict changes in δ^18^O_sw_ through Earth history show trends that asymptotically approach modern values, consistent with minimal change in carbonate δ^18^O over the past ~ 350 myr (e.g. Ref.^40^). One limitation of crustal exchange models is the large uncertainty in hydrothermal fluid fluxes. These fluxes are estimated based on the difference between modeled and measured (conductive) ocean heat flux^41^. However, uncertainty in the temperature of off-ridge hydrothermal fluids can lead to greater than ± 50% uncertainty in hydrothermal flux and large uncertainty in the mineral–water ^18^O fractionation. A recent hypothesis is that Snowball-Earth sequestration of glacial ice might have resulted in ^18^O-enriched residual seawater that would re-equilibrate with ocean crust, lowering δ^18^O_sw_ toward 0‰, followed by melting of ^18^O-depleted Snowball ice and lowering of global δ^18^O_sw_^42^. However, for this process to impact the δ^18^O of Cambrian and Ordovician oceans, large ice volumes with extremely low δ^18^O would be required in a slushball Earth^42^, an improbable scenario.

In summary, the different models for the temporal δ^18^O_sw_ trend each lead to extreme climate scenarios. Assumption of a crustally-buffered hydrosphere near −1‰ (VSMOW) leads to high paleotemperatures in the Early to Middle Ordovician (> 45 °C). In contrast, the Phanerozoic δ^18^O_sw_ trend of Veizer and Prokoph^4^ generates low-latitude δ^18^O temperatures of ~ 10 °C for the Late Ordovician, Pennsylvanian, and Early Cretaceous (Fig. 3 of Ref.^22^), temperatures incompatible with modern photozoan carbonate deposition mainly observed within the > 20 °C winter water temperature isotherm^43^.

Studies of the δ^18^O of non-carbonate phases, clumped isotopes in carbonates, and fluid inclusions support arguments for relatively high seawater δ^18^O and temperatures in the early Paleozoic. Hydrothermally-altered ophiolites^31^ and mudrocks^32^ yield constant δ^18^O values through time, suggesting constant seawater δ^18^O throughout the Phanerozoic. Moreover, magnetite veins in Moroccan ophiolites dated at 760 Ma indicate δ^18^O_sw_ values of −1.3 ± 1.0‰^44^. Further, δ^18^O values of marine iron oxides from ooidal ironstones and other deposits spanning the last 2 billion years suggest lower δ^18^O_sw_ in the Proterozoic but “largely stable δ^18^O_sw_ in the Phanerozoic”^45^. Clumped isotopes indicate high temperatures for the Ordovician and Silurian^23^. For example, temperatures for Katian (~ 450 Ma) brachiopods and rugose corals from North America cluster around ~ 37 °C, while minimum Hirnantian (~ 444 Ma) values range from 29 to 35 °C^46,47^. These temperatures and associated δ^18^O values suggest δ^18^O_sw_ values of −0.5 to 3.5‰. Reexamination of Katian brachiopods using microanalytical techniques yields clumped isotope temperatures with a mode of ~ 33 °C (mean = 35 ± 2.8 °C) for a subtropical upwelling setting^24^. These temperatures and carbonate δ^18^O values equate to a mean seawater δ^18^O of −0.3 ± 0.6‰ VSMOW. Tropical Silurian brachiopods yield similar clumped isotope temperatures and seawater δ^18^O values (33 ± 7 °C and −0.3 ± 1.3‰ respectively) for “the most pristine materials”^48^. Lastly, homogenization temperatures of fluid inclusions in Ediacaran halite (circa 546 Ma) also indicate high temperatures (39 °C^49^).

Another consideration in paleotemperature determination is paleoceanographic environment. The samples on which Paleozoic through Jurassic temperatures are based come from continental margins and epeiric seas, environments subjected to local runoff and restricted circulation. Local runoff can result in δ^18^O_sw_ values lower than those estimated here, but typically such cases can be identified by the presence of euryhaline fauna^50^. Restricted circulation, on the other hand, can lead to temperatures that average 2 °C higher than open-ocean temperatures^51^; therefore, the Paleozoic and some Mesozoic temperatures reported in this study may be 2 °C higher than those of the contemporaneous open ocean.

Extreme warmth comparable to the Late Ordovician to Devonian has been recorded in younger times in Earth history. For example, δ^18^O measurements of end-Permian and Early Triassic conodonts yield temperatures of ≥ 36 °C^9,10^. TEX_86_ and δ^18^O data for planktonic foraminifera suggest late Cenomanian-to-Turonian equatorial SSTs of ≥ 35 °C^16^. Furthermore, multiple methods (TEX_86_^H^-SST calibration, Mg/Ca, Δ_47_^17^) indicate tropical SSTs throughout the Eocene of 30 to 36 °C for the Paleocene-Eocene Thermal Maximum (PETM), and ~ 35 to ~ 37 °C for the Early Eocene Climatic Optimum (EECO^52^; Fig. 4). Even higher TEX_86_ SSTs of 40 to 45 °C are reconstructed using the TEX_86_ BAYSPAR calibration^16,53^. Thus, low-latitude SSTs of 35 °C and warmer are not unique to the Early Paleozoic but occurred also during Mesozoic and Cenozoic warm intervals. Furthermore, these temperatures are close to tropical SST projected for the year 2100 if modern tropical seas (25–30 °C) warm by more than 4 °C^54,55^, as predicted by the RCP 8.5 scenario.

Comparing low-latitude temperature to seafloor accretion rate^22^ allows us to examine the link between plate tectonics and climate. Seafloor accretion rate correlates significantly with low latitude temperature (R^2^ = 0.47, *p* < 0.0001; Supplementary Fig. S1, Supplementary Table S2), with warmer temperatures associated with faster spreading rates. This linkage is attributed to high rates of volcanic CO_2_ degassing with higher rates of seafloor spreading and subduction^30^.

Isotopic temperatures of > 40 °C for the late Cambrian and Early Ordovician, which extend beyond the temperature tolerances of most modern multi-cellular Eukarya (e.g. Ref.^56^), present a conundrum. Is our understanding of the physiology and behavior of early animals incomplete? If Early Ordovician fauna were limited only to taxa able to tolerate unusually high temperatures, this would further strengthen Ordovician cooling as an explanation for the dramatic increase in Ordovician diversity, the Great Ordovician Diversification Event (GOBE)^57^. Furthermore, such cooling would raise the solubility of oxygen in seawater^29^ and, along with a posited increase in atmospheric oxygen levels, permit greater metabolic activity and predation^58^, leading to paleoecological reorganization^59^.

**Figure 6:**
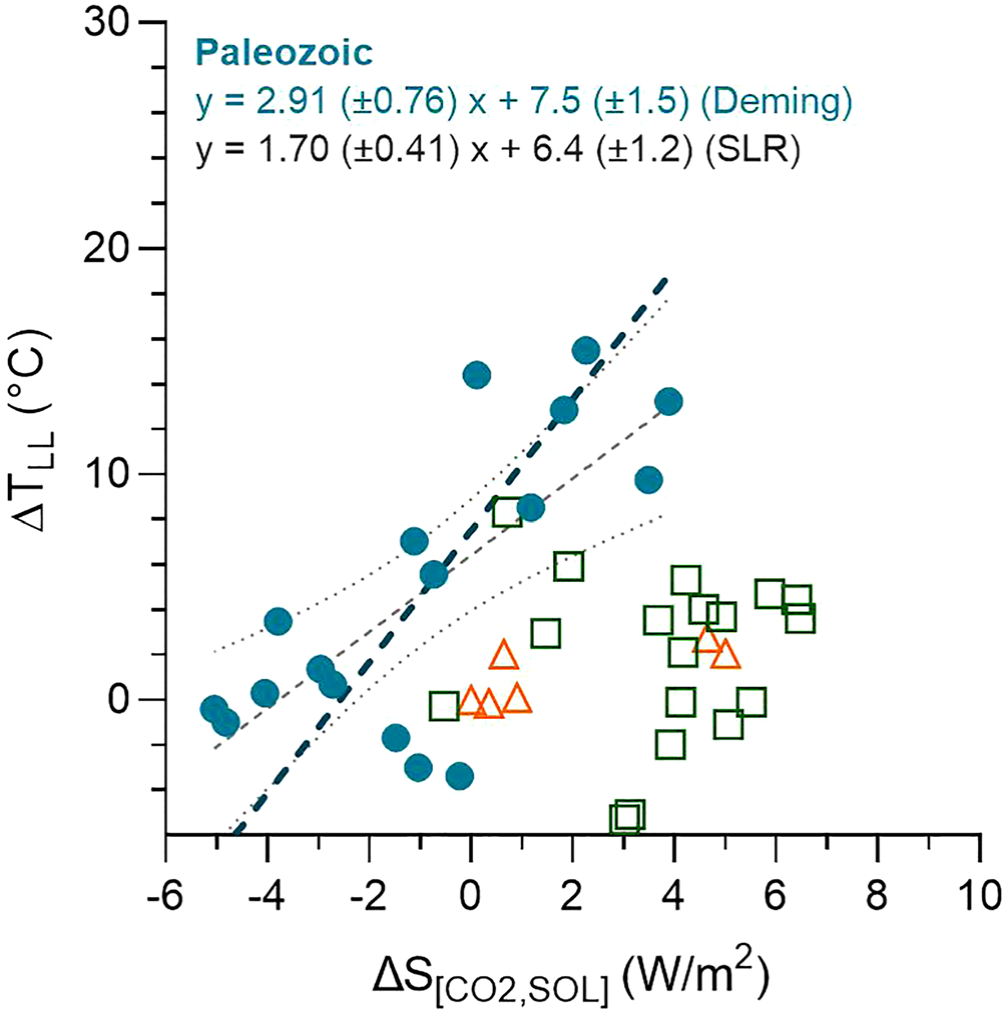
Change in *p*CO_2_ and solar radiative forcing (ΔS_[CO2,SOL]_) versus mean low-latitude (0–30°) paleotemperature (ΔT_LL_) for 10-myr intervals in the Cenozoic (orange), Mesozoic (green), and Paleozoic (blue-green). Filled symbols identify statistically significant relations (p < 0.05). Equations show Deming Model II regression (heavy dashed lines) and simple linear regression with associated uncertainty (gray dashed line with dotted uncertainty bands). Only Paleozoic data show a significant relationship.

Our results can be used to examine the sensitivity of low-latitude temperatures to changes in *p*CO_2_ (low-latitude Earth system sensitivity or ESS) Overall, paleotemperature exhibits a significant correlation (R^2^ = 0.234, *p* = 0.0014) with *p*CO_2_ doubling based on the proxy record of Foster et al.^21^; Supplementary Table S3; based on 10-myr Locfit averages). This relationship indicates a role of *p*CO_2_ in controlling low-latitude Phanerozoic SST. For Phanerozoic climate, changes in solar radiation must be considered in addition to the radiative forcing controlled by *p*CO_2_. To examine the relationship between changes in low-latitude temperature (ΔT_LL_) and changes in radiative forcing of *p*CO_2_ and solar radiation (ΔS_[CO2,SOL]_), we convert *p*CO_2_ doubling to radiative forcing by multiplying by 3.7 W m^−2^, and correct for increasing solar radiation with time using the equation: ΔF_sol_ = −238 W m^−2^ × 0.0000665 age (myr)^60^. ΔS_[CO2,SOL]_ estimates yield significant correlations (*p* < 0.0001) with ΔT_LL_ for the Paleozoic but not for the Mesozoic and Cenozoic (Fig. 6). Royer^61^ also found a poor relationship between surface temperature and combined CO_2_ and solar forcing for the Cenozoic and Mesozoic, noting that temperature data for the Mesozoic are sparse. Though not seen in our results based on limited planktonic foraminifera and macrofossil data, a strong relationship between Cenozoic temperatures and *p*CO_2_ is seen in studies based on benthic foraminiferal δ^18^O^62^.

Deming Model II (DMII) regression for the Paleozoic data generates the following relation:

2 $$\Delta {\text{T}}_{{{\text{LL}}}} \left( {^\circ {\text{C}}} \right) \, = { 2}.{91 }\left( { \pm 0.{76}} \right) \, \cdot \, \Delta {\text{S}}_{{[{\text{CO2}},{\text{ SOL}}]}} + { 7}.{5 }\left( { \pm {1}.{5}} \right) \, \left( {{\text{R}}^{{2}} = \, 0.{538}} \right).$$

DMII regression was chosen because of comparable uncertainty in both X and Y. This equation yields an Earth system sensitivity (ESS) value for Paleozoic low latitudes of 2.9 K W^−1^ m^2^ or 10.7 K per CO_2_ doubling. This value is high compared with the range for 35–150 Ma based on an ensemble of climate model simulations (3.5 and 5.5 K^63^), especially considering that (1) the 0°–30° latitude band accounts for only half of Earth’s surface and (2) SST change will underestimate change in global surface temperature, especially during icehouse climate^61,64^. On the other hand, the value is within those calculated for discrete time intervals in the Pliocene^61^. In comparing radiative forcing and temperature change, other studies of Earth system sensitivity have used simple linear regression (SLR), which considers only uncertainty in Y^61,65^. SLR of our results yields the following equation for the Paleozoic:

3 $$\Delta {\text{T}}_{{{\text{LL}}}} \left( {^\circ {\text{C}}} \right) \, = {1}.{7}0 \, \left( { \pm 0.{41}} \right) \, \cdot \, \Delta {\text{S}}_{{[{\text{CO2}},{\text{ SOL}}]}} + { 6}.{4 }\left( { \pm {1}.{2}} \right) \, \left( {{\text{R}}^{{2}} = \, 0.{538}} \right).$$

This equation provides an ESS value of 1.7 W^−1^ m^2^ or 6.3 K per CO_2_ doubling, similar to climate sensitivities determined for glaciated time^64^. Justification for using simple linear regression instead of Model II regression is discussed in Smith^66^ and centers on the objectives of the study. Since our objective is to “define some mutual, codependent “law” underlying the interaction between X [Δ*p*CO_2_ radiative forcing] and Y [ΔSST]”, and since the slope “will be used to interpret the pattern of change” (Smith^66^, p. 482), we favor Model II regression. On the other hand, Smith^66^ notes that “when an equation is used for prediction”, SLR is the “method of choice”. More detailed examination of choice of regression model is beyond the scope of this paper but clearly merits future consideration.

Note that this treatment does not account for changes in paleogeography, sea level, land ice area, vegetation, non-CO_2_ greenhouse gases, and aerosols, some of which also serve as feedback mechanisms; however, it does provide an estimate of ESS for the highest sustained CO_2_ levels in the past 420 myr (> 2000 ppm) and the worst-case scenario levels for a couple of centuries into the future^55^. Lastly, our finding that ESS was high in the Paleozoic compared with the Cenozoic supports studies suggesting higher ESS with higher CO_2_ levels (e.g. Ref.^67^).

## Materials and methods

Details regarding samples and methods appear in Ref.^1^. The compilation builds upon previous efforts (e.g. Refs.^4,27^) and focuses on carbonate and phosphate fossils and microfossils that (1) are widely distributed in the sedimentary record, (2) are precipitated with quantitative δ^18^O fractionation relative to temperature, and (3) exhibit excellent preservation. Samples include mollusks, brachiopods, planktonic foraminifera, fish teeth, and conodonts.

The late Cambrian through Triassic record is based on brachiopod calcite and conodont phosphate. Thick brachiopods from cratons tend to show the best preservation and least scatter in their δ^18^O values (e.g. Refs.^15,68,69^). Targeting best preserved shells with petrographic and cathodoluminescence microscopy, combined with analyses of microsamples (< 100 µg), further reduces variability and allows for multiple analyses from a single shell. While our compilation includes all data, regressions for temporal trends and paleotemperature estimates only consider data from brachiopod shell that (1) is non-luminescent, (2) contains manganese contents < 250 ppm, or (3) thick-shelled and from areas known for excellent preservation (e.g., Moscow Basin; see Ref.^1^ for additional details). Biogenic apatite is less prone to diagenetic overprinting; however, the unknown habitat of conodonts may represent an uncertainty for interpretation of δ^18^O values. While brachiopods are benthic organisms, conodonts were active swimmers that could have lived in warm surface waters or deeper and thus colder parts of the water column. The comparison of oxygen isotope values of conodont taxa from sediments of different water depths gave equivocal results (e.g. Refs.^70,71^).

Belemnite rostra, calcite deposits in the cephalopod’s posterior, are the most common material analyzed from Jurassic and Cretaceous sediments. These fossils typically have δ^18^O values higher than those of co-occurring bivalves, confounding paleotemperature studies. However, recent clumped isotope studies have revealed that belemnites precipitated in warmer waters than their δ^18^O values indicate, prompting researchers to conclude that belemnite guards are precipitated in true equilibrium with seawater, and that δ^18^O paleotemperature relations for other biominerals and laboratory precipitates do not represent true equilibrium^7,8^. In our dataset, belemnites average 1.7 ± 0.5‰ (N = 19) and 1.1 ± 1.2‰ (N = 13) higher in δ^18^O compared with brachiopods and bivalves (Appendix 4). To account for this effect, we have applied a −1.5‰ correction to belemnite data as suggested by Vickers et al.^8^.

Planktonic foraminiferal data provide paleotemperatures for Cretaceous through Cenozoic climate. Because planktonic foraminiferal tests commonly recrystallize with burial on the sea floor^34^, we only use data for planktonic foraminifera that exhibit exceptional preservation (e.g., “glassy”, “excellent”^16,34^). The δ^18^O data for aragonite samples, mostly of Cenozoic age, are normalized to calcite δ^18^O by subtracting 0.6‰^72^.

### Analytical techniques

Analytical techniques are summarized in Grossman and Joachimski^1^ and Joachimski et al.^70^ and presented in detail in the papers from which the data are derived. Briefly, carbonates of 0.05 to several milligrams are acidified with concentrated phosphoric acid and the CO_2_ evolved is analyzed on an isotope ratio mass spectrometer. Isotopic data are reported in delta (δ) notation and reported versus PDB (Peedee belemnite) or VPDB (Vienna PDB). The latter refers to calibration to PDB using the NBS-19 calcite standard (δ^18^O =  − 2.20‰ versus PDB^73,74^ or the new carbonate standard, IAEA-603 (δ^18^O =  − 2.37‰). The precision for oxygen isotope analyses of CaCO_3_ is typically ± 0.05 to 0.10‰, which equates to roughly ± 0.25 to ± 0.5 °C at 25 °C^12^. Oxygen isotope analyses of biogenic apatite are either measured by (1) TC-EA IRMS (thermally coupled elemental analyzer—isotope ratio mass spectrometry) on trisilverphosphate precipitated after dissolving calcium fluorapatite or (2) in situ by secondary ion mass spectrometry (SIMS). Whereas phosphate-bound oxygen is analyzed by TC-EA IRMS, total oxygen including phosphate-, carbonate- and hydroxyl-bound oxygen is measured by SIMS with the δ^18^O offset between these methodologies not well constrained. We applied a correction of −0.6‰ to all SIMS δ^18^O data based on the comparison of SIMS and TC-EA IRMS data^75^.

### Paleotemperature and seawater δ^18^O determinations

We use the Kim and O’Neil^12^ and Pucéat et al.^13^ δ^18^O paleotemperature equations for calcite and phosphate, respectively. The δ^18^O of seawater (δ^18^O_sw_) for million-year intervals is based on estimates of the volume and δ^18^O of glacial ice (see Supplementary Materials, Supplementary Tables S4–S6). Ice volumes through time are binned into simple categories of ice-free, low, moderate, and high based on studies of glacial sediments and sea level (e.g. Refs.^76–79^). For the δ^18^O of ice, we assume the δ^18^O values for the West Antarctica (−41‰) and Greenland ice sheets (−34‰) for “moderate” and “low” ice volumes respectively (Supplementary Table S4–S6). The calculated values for mean δ^18^O_sw_ range from −1.08‰ for the ice-free state to 0.45‰ for high ice volume (Pleistocene average; Supplementary Table S7). Lastly, δ^18^O_sw_ was averaged for 1-myr steps using a 2-myr window to smooth the impact of assigned ice volume changes.

### Paleolatitudinal correction

Paleolatitudes were reconstructed using the GPlates software^80^ with the Paleomap^81^ rotation model. For paleolatitudinal corrections of δ^18^O_sw_ during icehouse climates, we use the modern latitude-δ^18^O relationship of Roberts et al.^82^; Supplementary Fig. S2, Supplementary Table S8) derived from gridded data modeled in LeGrande and Schmidt^83^. Using data for the Southern Hemisphere, which are less influenced by landmasses than Northern Hemisphere data, yields the relationship for 0–60° latitude:

4 $$\Delta^{18} {\text{O}}_{{\text{sw,lat,Mod}}} = \, - 6.650 \, \times \, 10^{ - 4} \lambda^{2} + \, 3.363 \times \, 10^{ - 2} \lambda + \, 0.174 \, \left( {{\text{R}}^{{2}} \, = \, 0.951} \right).$$

For hothouse climates, we generate the δ^18^O_sw_-latitude relation using the isotope-enabled ocean–atmosphere general circulation model (GCM) results of Ref.^82^ (Supplementary Table S8) for the early Paleogene. The modeled δ^18^O_sw_ values for 0–60° latitude in the southern hemisphere (from Fig. 1 of Ref.^82^) give the equation:

5 $$\Delta^{18} {\text{O}}_{{{\text{sw}},{\text{lat}},{\text{Pg}}}} = \, - {4}.{944 } \times { 1}0^{{ - {4}}} \lambda^{{2}} + { 2}.{492} \times { 1}0^{{ - {2}}} \lambda + \, 0.{1}0{2 }\left( {{\text{R}}^{{2}} \, = \, 0.{932}} \right).$$

Latitudina﻿l corrections for < 60° latitude (δ^18^O_lat corr_) were proportioned based on the estimated δ^18^O_sw,iceV corr_ using the equation:

6 $${\Delta }^{18}{O}_\text{lat corr}={\Delta }^{18}{O}_{sw,lat,Mod} \frac{{\updelta }^{18}{O}_{sw,iceV} - -1.08}{(0.45 - -1.08)}+{\Delta }^{18}{O}_{sw,lat,Pg}\left(1-\frac{{\updelta }^{18}{O}_{sw,iceV} - -1.08}{(0.45 - -1.08)}\right)$$

where δ^18^O_sw,iceV_ is the ice volume correction for global seawater and 0.45‰ is the icehouse endmember (average between glacial and interglacial states).

We bin our data into the following climate zones by paleolatitude: tropical (± 10°), tropical-subtropical (10°–30°), temperate (30°–50°), and subpolar–polar (50°–90°) using the maps from Scotese and Wright^81^. These bins were selected based on the temperature gradients for the latest Cretaceous through Recent reported in Zhang et al.^84^. Over the time interval studied, spanning greenhouse and icehouse climates, paleotemperatures within 10° N or S are invariant with paleolatitude. Inflections in latitudinal temperature gradients at 30° and 50° define boundaries for the next two bins. Lastly, stage-averaged paleotemperatures for the ± 10° and 10°–30° bins were found to be statistically similar (ΔT(10°–30° minus 0°–10°) (°C) = −1.0 ± 2.1(2SE) °C) (N = 25) for carbonates and −2.0 ± 2.2 (N = 25) for phosphates) and thus were combined. At latitudes higher than 30°, data become sparse. Further, the increased latitudinal temperature gradient at higher latitudes along with greater influence of ^18^O-depleted fresh water^83^ increases the uncertainty in paleotemperature determinations. Lastly, sample ages are based on the GTS2020 timescale^85^.

## Supplementary Information

Supplementary Information 1.

Supplementary Information 2.

Supplementary Information 3.

Supplementary Information 4.

Supplementary Information 5.

Supplementary Information 6.

Supplementary Information 7.

Supplementary Information 8.

Supplementary Information 9.

## Data Availability

All data and Locfit regression tables used in this study are available in the Supplementary tables and auxiliary data files. The data are also available on the StabisoDB online database (http://stabisoDB.org).
