# Supplementary material for: Ocean temperatures through the Phanerozoic reassessed
Source: Sci Rep. 2022 May 27;12:8938. doi: 10.1038/s41598-022-11493-1 (PMC9142518; doi:10.1038/s41598-022-11493-1)
Supplement: Supplementary file 5 — Supplementary Information 5. [file 41598_2022_11493_MOESM5_ESM.docx]

Ocean Temperatures through the Phanerozoic Reassessed

*Short title: Phanerozoic Ocean Temperatures*

**Authors**

Ethan L. Grossman,^1*^ and Michael M. Joachimski^2^

**Affiliations**

*^1^Department of Geology and Geophysics, Texas A&M University, College Station, TX, USA 77843*

*^2^GeoZentrum Nordbayern, Friedrich-Alexander University of Erlangen-Nuremberg (FAU), Schlossgarten 5, 91054 Erlangen, Germany*

^*^ Corresponding author: e-grossman@tamu.edu

**Supplemental Materials**

**Seawater δ^18^O Determinations**

On less than tectonic time scales, seawater δ^18^O (δ^18^O_sw_) can vary with ice volume and local water mass, which in turn respond to global and local climate, drainage, and ocean circulation. As a first approximation, we calculate δ^18^O_sw_ for million-year intervals from estimates of the volume and δ^18^O of glacial ice, and from independent measures of paleotemperature change. Ice volumes are binned into simple categories of ice-free, low, moderate, and high (Table S4-S6). For the Early Paleozoic, ice volumes are estimated based on the studies of ^1, 2, 3, 4^. Ice volumes for the Late Paleozoic rely on the studies and compilations of ^5, 6,^ ^7, 8^. Lastly, Cenozoic ice volumes are approximated from sea level curves (e.g. ^9^).

For the Late Paleozoic, we assign maximum ice volume starting in the Bashkirian with culmination of a major ice sheet in the Asselian. We adopt the model of multiple smaller ice sheets during this period, and the estimate of 20 x 10^6^ km^3^ for maximum glaciation ^8^, which is “moderate” compared with modern ice volume. This is lower than earlier estimates (e.g., 40 x 10^6^ km^3^, ^10^, smallest scenario [ICE I]). To calculate the impact of ice volume on δ^18^O_sw_, the δ^18^O of ice must also be estimated. Smaller ice sheets should be associated with reduced latitudinal temperature gradients and thus precipitation with higher δ^18^O (e.g. ^11^); thus, the impact of ice volume on δ^18^O_sw_ will be further diminished. This is addressed by assuming the δ^18^O values for the West Antarctica (-41‰) and Greenland ice sheets (-34‰) for “moderate” and “low” ice volumes respectively (Table S4-S6). The calculated values for mean seawater δ^18^O range from -1.08‰ for the ice-free state to 0.45‰ for high ice volume (Pleistocene average; Table S7). For much of the Cenozoic, glacial ice volume as indicated by sea level ^9^ was “moderate”. We assign a δ^18^O_sw_ value of -0.48‰ for 34-0 Ma, which is in good agreement with the average δ^18^O_sw_ calculated from benthic foraminiferal δ^18^O and Mg/Ca ratios (-0.53 ±0.22‰; ^12^). Lastly, seawater δ^18^O is averaged for 1-myr steps using a 2-myr window to smooth the impact of assigned ice volume changes (Table S7). The methods for determining latitudinal dependence of seawater δ^18^O are discussed in the text.

Our treatment does not consider the influences of continental configuration, runoff, ocean circulation, and local water masses on seawater δ^18^O. Furthermore, the δ^18^O-latitude models do not account for uncertainties in model parameters such as *p*CO_2_, CO_2_ climate sensitivity, and paleogeography. Despite the uncertainties, quantitation of the effects of ice volume and latitude corrections on seawater δ^18^O should improve the accuracy of tropical and subtropical isotopic temperatures, especially in low latitudes (30°S to 30°N) less influenced by high-latitude runoff and glacial meltwater. Differences between model results (e.g. ^13^ vs. ^14^) highlight the need for more careful evaluation, and isotope-enabled climate models using Paleozoic paleogeographies.

Solution pH has been shown to correlate inversely with oxygen isotopic fractionation in some organisms presumably due to incorporation of ^18^O-enriched aqueous CO_2_ into the shell ^15, 16, 17, 18^. This should result in higher δ^18^O values during intervals with high *p*CO_2_ and high temperature, which implies high δ^18^O temperatures recorded in this and other studies may be underestimated. However, pH effects are inconsistent and decrease with increasing temperature ^19^. Thus, the effect of low pH on δ^18^O during high-*p*CO_2_ greenhouse climates may be ameliorated by high temperatures. Another consideration, Paleozoic ocean pH is poorly constrained because the best method for this determination, the boron isotope method, is complicated by variation in the boron isotopic composition of Paleozoic seawater ^20, 21^. Consequently, no adjustment has been made for the effect of pH on the oxygen isotope composition of carbonates in this study.

**Error Analysis**

The errors associated with the paleotemperature determinations are threefold: (1) measurement error, (2) error in the paleotemperature equation and its adherence by the sample material (i.e., vital effect), and (3) error in the estimation of the δ^18^O of the waters of precipitation (δ^18^O_sw_). The last, estimation of δ^18^O_sw_, requires determination of the global average value, latitudinal variation associated with the hydrologic cycle, and local effects. These errors will vary as a function of sample material and in some cases, age. Thus, different errors have been estimated for carbonate and phosphate minerals, and for Cenozoic, Mesozoic, and Paleozoic samples.

**Measurement error (s_m_).** Analytical precision for δ^18^O measurements of carbonates and phosphates is better than 0.1‰ and 0.3‰ respectively in most labs. Using the slopes of the Kim and O’Neil ^22^ and Pucéat et al. ^23^ equations as a function of temperature (dT/dδ^18^O = -4.92 and -4.22 °C/‰), these equate to uncertainties of 0.5 °C and 1.3 °C (Table S9).

**Paleotemperature equation (s_pe_)**. The error used is the standard deviation of the residuals for the regression. For the linearized version of the Kim and O’Neil ^22^ equation:

T (°C) = 163.5 (±3.37) – 4.92 (±0.121) · 1000lnα

this value is 0.8 °C. For Pucéat et al.^23^ the value is 2.7 °C.

**Ice volume effect on δ^18^O_sw_ (s_iv_).** This is evaluated based on the average difference between estimated δ^18^O_sw_ from ice volume for the Cenozoic and estimated δ^18^O_sw_ from Mg/Ca ratios of benthic foraminifera ^12^).

**Paleolatitude effect on δ^18^O_sw_ (s_pl_)** The standard deviations of Paleogene and Modern Δ^18^O_sw_ values (deviation from mean δ^18^O_sw_) for 0-30° latitude at 5° intervals are only 0.33‰ and 0.47‰. To calculate uncertainty we use is the standard deviation of the absolute difference between the two models for 0 - 30° latitude at 5° intervals (±0.07‰ or ±0.35 °C and ±0.30 °C for carbonates and phosphates respectively). Considering the small error associated with the dependence of Δ^18^O_sw_ on paleolatitude, no attempt is made to account for errors in paleolatitude.

**Local δ^18^O_sw_** (s_lc_). The deviation of local seawater δ^18^O in a restricted basin from that of global seawater at that latitude is arguably the largest source of uncertainty in oxygen isotope paleothermometry. In Paleozoic through early Cretaceous samples, this uncertainty is amplified by the preponderance of older samples (>115 Ma) from epeiric seas. Restricting the paleotemperature curve to latitudes less than 30° reduces, but does not eliminate, the influence of low-δ^18^O freshwater input. For the modern ocean, a reasonable salinity (S) range (95% or 2 x standard deviation σ) for nearshore conditions habitable by stenohaline fauna like brachiopods is 32-38 psu. Using the average Δδ^18^O_sw_/ΔS of the Caribbean coast of Panama, which experiences relatively high salinities and input of Cordilleran-fed freshwater of low δ^18^O (-5.75‰ VSMOW), yields a δ^18^O range of 0.19 ‰/psu x 6 psu or 1.14‰ ^24^. Accepting that this range is 2 x ±2σ, the ±1σ uncertainty is ±0.3‰. This value matches the standard deviation for a 112-analysis grid across a sectioned Pennsylvanian brachiopod shell ^25^.

Total error is calculated by taking the square root of the squares of each uncertainty component: Σs = (s_m_^2^ + s_pe_^2^ + s_iv_^2^ + s_pl_^2^ + s_lc_2)^0.5^. Estimated paleotemperature error averages ±2.0 °C for carbonates and ±2.7 °C for phosphates (Table S9). Pooling of data in the Locfit regression reduces the error associated with individual measurements. Uncertainty in the Locfit regression model varies with sample density, ranging from 0.25 to 1.35 °C where sufficient data were available. For intervals with little or no data, regressions are interrupted.

References

1. Came RE, Eiler JM, Veizer J, Azmy K, Brand U, Weidman CR. Coupling of surface temperatures and atmospheric CO_2_ concentrations during the Palaeozoic era. *Nature* **449**, 198-U193 (2007).

2. Eyles N. Glacio-epochs and the supercontinent cycle after ∼3.0 Ga: Tectonic boundary conditions for glaciation. *Palaeogeogr., Palaeoclimat., Palaeoecol.* **258**, 89-129 (2008).

3. Ghienne J-F*, et al.* A Cenozoic-style scenario for the end-Ordovician glaciation. *Nat. Commun.* **5**, 4485 (2014).

4. Creveling JR, Finnegan S, Mitrovica JX, Bergmann KD. Spatial variation in Late Ordovician glacioeustatic sea-level change. *Earth Planet. Sci. Lett.* **496**, 1-9 (2018).

5. Isbell JL, Miller MF, Wolfe KL, Lenaker PA. Timing of late Paleozoic glaciation in Gondwana:Was glaciation responsible for the development of northern hemisphere cyclothems? In: *Extreme depositional environments: Mega end members in geologic time* (eds Chan MA, Archer AW). (Geological Society of America, 2003).

6. Fielding CR, Frank TD, Isbell JL. The late Paleozoic ice age-A review of current understanding and synthesis of global climate patterns. In: *Resolving the Late Paleozoic Ice Age in Time and Space* (eds Fielding CR, Frank TD, Isbell JL) 343-354 (Geological Society of America*,* 2008).

7. Isbell JL*, et al.* Glacial paradoxes during the late Paleozoic ice age: Evaluating the equilibrium line altitude as a control on glaciation. *Gondwana Res.* **22**, 1-19 (2012).

8. Montañez IP, Poulsen CJ. The Late Paleozoic ice age: An evolving paradigm. *Annual Rev. Earth Planet. Sci.* **41**, 629-656 (2013).

9. Miller KG, Browning JV, Schmelz WJ, Kopp RE, Mountain GS, Wright JD. Cenozoic sea-level and cryospheric evolution from deep-sea geochemical and continental margin records. *Sci. Adv.* **6**, eaaz1346 (2020).

10. Crowley TJ, Baum SK. Estimating Carboniferous sea-level fluctuations from Gondwanan ice extent. *Geology* **19**, 975-977 (1991).

11. Gasson E, DeConto RM, Pollard D, Levy RH. Dynamic Antarctic ice sheet during the early to mid-Miocene. *Proceed.Nat. Acad. Sci.* **113**, 3459-3464 (2016).

12. Lear CH, Elderfield H, Wilson PA. Cenozoic deep-sea temperatures and global ice volumes from Mg/Ca in benthic foraminiferal calcite. *Science* **287**, 269-272 (2000).

13. Roberts CD, LeGrande AN, Tripati AK. Sensitivity of seawater oxygen isotopes to climatic and tectonic boundary conditions in an early Paleogene simulation with GISS ModelE-R. *Paleoceanography* **26**, (2011).

14. Tindall J, Flecker R, Valdes P, Schmidt DN, Markwick P, Harris J. Modelling the oxygen isotope distribution of ancient seawater using a coupled ocean–atmosphere GCM: Implications for reconstructing early Eocene climate. *Earth Planet. Sci. Lett.* **292**, 265-273 (2010).

15. Spero HJ, Bijma J, Lea DW, Bemis BE. Effect of seawater carbonate concentration on foraminiferal carbon and oxygen isotopes. *Nature* **390**, 497-500 (1997).

16. Beck WC, Grossman EL, Morse JW. Experimental studies of oxygen isotope fractionation in the carbonic acid system at 15 degrees, 25 degrees, and 40 degrees C. *Geochim. Cosmochim. Acta* **69**, 3493-3503 (2005).

17. Zeebe RE. An expression for the overall oxygen isotope fractionation between the sum of dissolved inorganic carbon and water. *Geochem. Geophys. Geosyst.* **8**, 7 (2007).

18. Ye F*, et al.* Variation in brachiopod microstructure and isotope geochemistry under low-pH–ocean acidification conditions. *Biogeosciences* **16**, 617-642 (2019).

19. Devriendt LS, Watkins JM, McGregor HV. Oxygen isotope fractionation in the CaCO3-DIC-H2O system. *Geochim. Cosmochim. Acta* **214**, 115-142 (2017).

20. Joachimski MM, Simon L, van Geldern R, Lécuyer C. Boron isotope geochemistry of Paleozoic brachiopod calcite: Implications for a secular change in the boron isotope geochemistry of seawater over the Phanerozoic. *Geochim. Cosmochim. Acta* **69**, 4035-4044 (2005).

21. Legett SA, Rasbury ET, Grossman EL, Hemming NG, Penman DE. The brachiopod δ^11^B record across the Carboniferous-Permian climate transition. *Paleoceanogr. Paleoclimatol.* **35**, e2019PA003838 (2020).

22. Kim ST, O'Neil JR. Equilibrium and nonequilibrium oxygen isotope effects in synthetic carbonates. *Geochim. Cosmochim. Acta* **61**, 3461-3475 (1997).

23. Pucéat E*, et al.* Revised phosphate-water fractionation equation reassessing paleotemperatures derived from biogenic apatite. *Earth Planet. Sci. Lett.* **298**, 135-142 (2010).

24. Tao K, Robbins JA, Grossman EL, O'Dea A. Quantifying Upwelling and Freshening in Nearshore Tropical American Environments Using Stable Isotopes in Modern Gastropods. *Bull. Mar. Sci* .**89**, 815-835 (2013).

25. Mii HS, Grossman EL. Late Pennsylvanian seasonality reflected in the ^18^O and elemental composition of a brachiopod shell. *Geology* **22**, 661-664 (1994).

26. LeGrande AN, Schmidt GA. Global gridded data set of the oxygen isotopic composition in seawater. *Geophys. Res. Lett.* **33**, (2006).

27. Vérard C, Veizer J. On plate tectonics and ocean temperatures. *Geology* **47**, 881-885 (2019).

28. Poore RZ, Williams RS, Jr., Tracey C. Sea level and climate. In: *U.S. Geological Survey Fact Sheet 002–00*). (U.S. Geological Survey, 2000).

29. Eakins BW, Sharman GF. Volumes of the World's Oceans from ETOPO1. (National Geophysical Data Center, NOAA, 2010).

30. Lhomme N, Clarke GKC, Ritz C. Global budget of water isotopes inferred from polar ice sheets. *Geophys. Res. Lett.* **32**, (2005).

**Supplementary Materials**

**Figures**

Figure S1. Comparison of select carbonate δ^18^O versus global mean accretion rate (AccR) and global mean age of the sea floor (OcF Age). AccR and OcF ages from ^20^.

Figure S2. Seawater δ^18^O versus southern hemisphere latitude. Digitized data used for determining seawater δ^18^O versus latitude for icehouse (A) and greenhouse (B) climates (Table S7; from ^26^ in ^13^).

**Tables**

Table S1. Summary of mean oxygen isotope temperatures by stage for Paleozoic carbonates and phosphates. (See separate file.)

Table S2. Comparison of oxygen isotopic values of select carbonates and global mean age of the sea floor (OcF Age) and global mean accretion rate (AccR). (See separate file.)

Table S3. Values of temperature, temperature change, pCO_2_, and pCO_2_ and solar forcing with errors. (See separate file.)

Table S4. Volumes and δ^18^O (‰ VSMOW) of modern water used for estimate of seawater δ^18^O through time.

Table S5. Estimates of different reservoirs and their isotopic composition.

Table S6. Estimate of δ^18^O of seawater at different glacial states.

Table S7. Estimates of seawater δ^18^O (δ^18^O_sw_). (See separate file.)

Table S8. Digitized data for determining seawater δ^18^O versus latitude.

Table S9. Errors estimated for paleotemperature determinations.

Auxiliary Files

Appendix 1. Oxygen and carbon isotopic compositions of biogenic carbonates and sample information (GrossmanJoachimski_ SR_App-1-CaCO3-v1.1.xlsx).

Appendix 2. Oxygen isotope compositions of biogenic phosphates and sample information (GrossmanJoachimski_ SR_App-2-PO4-v1.1.xlsx).

Appendix 3. Count of oxygen isotope analyses by taxonomic group and climate zone.

Appendix 4. Comparison of δ^18^O of Jurassic and Cretaceous taxonomic groups by stage.

**Figures**

**Figure S1.**  Comparison of low-latitude temperature versus global mean accretion rate (AccR) and global mean age of the sea floor (OcF Age). AccR and OcF Age from ^27^. Lines and equations are derived from simple linear regression.

**Figure S2.** Seawater δ^18^O versus southern hemisphere latitude. Digitized data used for determining seawater δ^18^O versus latitude for icehouse (A) and greenhouse (B) climates (Table S7; from ^26^ in ^13^).

**Table S4. Volumes and δ^18^O (‰ VSMOW) of modern ice used for estimate of seawater δ^18^O through time.**

| Location | Volume (10^6^ km^3^)† | Mass (10^18^ kg, 920 kg/m^3^ for ice) | Potential sea-level rise, (m) | % mass | Average δ^18^O of ice§ | fraction of total *δ^18^O_ice_ | Effect on δ^18^O_sw_ |
| --- | --- | --- | --- | --- | --- | --- | --- |
| East Antarctic ice sheet | 26.04 | 23.96 | 64.8 | 1.71% | -56.5 | -45.5 | -0.97 |
| West Antarctic ice sheet | 3.26 | 3.00 | 8.06 | 0.21% | -41.0 | -4.1 | -0.09 |
| Antarctic Peninsula | 0.23 | 0.21 | 0.46 | 0.01% | -22.0 | -0.2 | 0.00 |
| Greenland | 2.62 | 2.41 | 6.55 | 0.17% | -34.0 | -2.8 | -0.06 |
| All other ice caps, ice fields, and valley glaciers | 0.18 | 0.17 | 0.45 | 0.01% | -20.0 | -0.1 | 0.00 |
| Total ice | 32.33 | 29.74 | 80.32 | 2.13% |  | -52.7 | **-1.12** |
| Ocean (volume and mass of water) | 1,335 | 1,368 |  | 97.87% |  |  |  |
| Total hydrosphere (excluding ground and formation waters) | 1,367 | 1,398 |  | -1.08 |  |  |  |

†^28^, ‡^29^, §^30^

**Table S5. Estimates of different water reservoirs and their isotopic composition (‰ VSMOW).**

| Reservoir | δ^18^O (‰) | Source |  |
| --- | --- | --- | --- |
| Glacial ice |  |  |  |
| High (modern) | -52.4 | Global ice sheet mass balance | |
| Moderate | -41 | East Antarctica |  |
| Low | -34 | Greenland |  |
| Ocean water | 0.04 | (**^26^**) | |
| Modern hydrosphere | **-1.08** | Assumes low δ^18^O of fresh groundwater balanced by higher δ^18^O of saline formation waters. | |

**Table S6.** **Estimate of δ^18^O of seawater at different glacial states.**

| Relative ice volume | Ice volume (10^6^ km^3^) | Mass (10^18^ kg, 920 kg/m^3^) | Calculated δ^18^O_sw_ | Cenozoic analogs | Source | |  |  | |  |  |
| --- | --- | --- | --- | --- | --- | --- | --- | --- | --- | --- | --- |
| Ice free | **0** | 0.00 | **-1.08** | Early Eocene | (^12^) | |  |  | |  |  |
| Low | **10** | 9.20 | **-0.83** | Mid Miocene | (^12^) | |  |  | |  |  |
| Moderate | **20** | 18.40 | **-0.48** | Oligocene | (^12^) | |  |  | |  |  |
| High | **40** | 36.80 | **0.45** |  |  | |  |  | |  |  |
| Modern | **32.33** | 29.74 | **0.04** | Modern | (^26^) | |  |  | |  |  |
| *Assumes no change in the volume and δ^18^O of waters from the terrestrial subsurface | | | | | |  | | |  | |  |
| ^Assumes no change in the volume and δ^18^O of waters from the marine sediment subsurface | | | | | | | | |  | |  |

.

**Table S8. Digitized data for determining seawater δ^18^O versus latitude.**

A. Modern (Southern Hemisphere). Digitized data of ^26^ in ^13^.

| Latitude | Lat relative to meteorological Equator | Absolute (meteor. Equator) | δ^18^O_sw_ (‰) | δ^18^O_sw, calc_ |
| --- | --- | --- | --- | --- |
| -69.12 | -74.12 | 74.12 | -0.33 | -0.21 |
| -64.68 | -69.68 | 69.68 | -0.33 | -0.34 |
| -59.30 | -64.30 | 64.30 | -0.31 | -0.33 |
| -55.79 | -60.79 | 60.79 | -0.25 | -0.25 |
| -51.35 | -56.35 | 56.35 | -0.14 | -0.10 |
| -47.84 | -52.84 | 52.84 | -0.01 | 0.04 |
| -45.26 | -50.26 | 50.26 | 0.13 | 0.14 |
| -43.16 | -48.16 | 48.16 | 0.26 | 0.22 |
| -39.18 | -44.18 | 44.18 | 0.38 | 0.37 |
| -35.44 | -40.44 | 40.44 | 0.50 | 0.48 |
| -32.16 | -37.16 | 37.16 | 0.58 | 0.56 |
| -28.19 | -33.19 | 33.19 | 0.66 | 0.62 |
| -24.21 | -29.21 | 29.21 | 0.66 | 0.64 |
| -20.47 | -25.47 | 25.47 | 0.58 | 0.63 |
| -16.49 | -21.49 | 21.49 | 0.52 | 0.58 |
| -12.75 | -17.75 | 17.75 | 0.47 | 0.52 |
| -8.54 | -13.54 | 13.54 | 0.45 | 0.44 |
| -4.33 | -9.33 | 9.33 | 0.44 | 0.36 |
| 0.12 | -4.88 | 4.88 | 0.35 | 0.31 |
| 3.63 | -1.37 | 1.37 | 0.25 | 0.31 |
|  |  |  |  |  |
|  |  |  |  |  |

B. Paleogene (Southern Hemisphere)^13^. Atmospheric concentrations of CO_2_ and CH_4_ were set at 4 × preindustrial CO_2_ and 7 × preindustrial CH_4_ (equivalent to ∼4.3 × preindustrial CO_2_).

| Latitude (°S) | δ^18^O_sw_ | δ^18^O_sw, calc_ | Latitude (°S) | δ^18^O_sw_ | δ^18^O_sw, calc_ |
| --- | --- | --- | --- | --- | --- |
| 64.93 | -0.22 | -0.38 | 22.39 | 0.48 | 0.47 |
| 57.14 | -0.07 | -0.05 | 18.36 | 0.47 | 0.45 |
| 51.72 | 0.04 | 0.04 | 15.24 | 0.42 | 0.41 |
| 60.76 | -0.15 | -0.16 | 12.88 | 0.36 | 0.36 |
| 44.91 | 0.15 | 0.13 | 9.83 | 0.28 | 0.29 |
| 38.44 | 0.25 | 0.25 | 6.50 | 0.20 | 0.21 |
| 30.31 | 0.37 | 0.40 | 3.52 | 0.15 | 0.14 |
| 25.59 | 0.45 | 0.46 | 0.81 | 0.11 | 0.11 |

**Table S9. Errors estimated for paleotemperature determinations.**

|  | Cenozoic | Mesozoic | Paleozoic | Cenozoic | Mesozoic | Paleozoic |
| --- | --- | --- | --- | --- | --- | --- |
|  | CaCO_3_ δ^18^O | | | CaCO_3_ temperature (°C) | | |
|  |  |  |  |  |  |  |
| Measurement | 0.1 | 0.1 | 0.1 | 0.5 | 0.5 | 0.5 |
| Ice volume | 0.13 | 0 | 0.26 | 0.65 | 0 | 1.3 |
| Paleolatitude | 0.07 | 0.07 | 0.07 | 0.35 | 0.35 | 0.35 |
| Local δ^18^O_sw_ | 0.3 | 0.3 | 0.3 | 1.5 | 1.5 | 1.5 |
| Paleotemperature equation |  |  |  | 0.8 | 0.8 | 0.8 |
| **Total uncertainty (square root of sums of squares)** |  |  |  | **1.9** | **1.8** | **2.2** |
|  |  |  |  |  |  |  |
|  | PO_4_ δ^18^O | | | PO_4_ temperature (°C) | | |
| Measurement | 0.3 | 0.3 | 0.3 | 1.3 | 1.3 | 1.3 |
| Ice volume | 0.13 | 0 | 0.26 | 0.5 | 0.0 | 1.1 |
| Paleolatitude | 0.07 | 0.07 | 0.07 | 0.3 | 0.3 | 0.3 |
| Local δ^18^O_sw_ | 0.3 | 0.3 | 0.3 | 1.3 | 1.3 | 1.3 |
| Paleotemperature equation |  |  |  | 2.7 | 2.7 | 2.7 |
| **Total uncertainty (square root of sums of squares)** |  |  |  | **3.3** | **3.3** | **3.4** |
